# Liver Segment and Lesion Segmentation on CT and MRI: An Open-Source Contribution to TotalSegmentator

**DOI:** 10.1007/s10278-025-01716-y

**Published:** 2025-10-24

**Authors:** Andrew Phillip Nicoli, Michael Bach, Jakob Wasserthal, Ashraya Kumar Indrakanti, Martin Segeroth, Shan Yang, Joshy Cyriac, Daniel Boll, Adrian Jonathan Wilder-Smith

**Affiliations:** Clinic of Radiology and Nuclear Medicine, University Hospital Basel, Petersgraben 4, 4031 Basel, Switzerland

**Keywords:** Automated liver segment segmentation, Liver lesions segmentation, Liver lesion detection, Multimodal, NnU-Net, TotalSegmentator

## Abstract

This study aims to develop a tool based on deep learning algorithms for automatic liver segment and liver lesion segmentation on Computed Tomography (CT) and Magnetic Resonance Imaging (MRI). We demonstrate its clinical utility using a qualitative example of hepatocellular carcinoma (HCC) response to transarterial chemoembolization (TACE). The models are provided as open-source software to update and improve the capabilities of TotalSegmentator. Liver segmentation was performed on 193 CTs and 120 MRIs, using fivefold cross-validation for training/testing. 429 CTs and 321 MRIs with liver lesions and 15 CTs and 13 MRIs without liver lesions were collected. Of these 414 CTs and 308 MRIs were manually segmented and a nnU-Net was trained on 750 images and tested on 80. Inter-rater variability was examined on 20 CTs and 20 MRIs by two independent readers. We analyzed its potential clinical utility on 172 TACE-treated HCC on CT. Performance was evaluated using sensitivity, false positives, and volume. Voxel-wise segmentation was evaluated using the Dice coefficient. Our model’s liver segmentation achieved Dice coefficients of 0.897 for CT and 0.847 for MRI. Liver lesion detection on CT achieved 75.8% sensitivity, 0.522 false positives per case (FP/c), and 0.658 Dice; on MRI, 62.7% sensitivity, 1.029 FP/c, and 0.337 Dice. Following TACE, median HCC attenuation significantly decreased from 51.33 HU to 38.5 HU. Human readers showed higher agreement (sensitivity: 64.7%, Dice: 0.464) than Lesion model (LM)-reader comparisons (sensitivity: 53.2%, Dice: 0.432) and the LM had a slightly higher FP/c (0.825 vs. 0.775). Overall, our algorithms reliably detect and segment liver segments and lesions on both CT and MRI and the qualitative assessment of HCC response to TACE illustrates the model’s potential value for clinical and research applications.

## Introduction

Follow-up imaging of liver lesions is becoming increasingly common due to advances in both surgical and medical management of liver malignancies [1]. This has led to longer follow-up periods and more complex pathology, including cases with multiple lesions, post-operative changes, and post-treatment modifications such as those seen with loco-regional therapies. Accurate interpretation of these studies often requires careful comparison of lesion size and morphology over time, which can be tedious and time-consuming. The workload is further compounded when multiple lesions must be tracked across different imaging modalities and timepoints. In such scenarios, radiologists would benefit from an automated tool capable of detecting, segmenting, and measuring liver lesions, as well as providing anatomical localization within Couinaud liver segments—thereby streamlining reporting and increasing clinical efficiency. Segment-level localization also standardizes longitudinal follow-up and reporting when multiple lesions must be tracked across modalities and timepoints.

Recent advances in convolutional neural networks (CNNs) have demonstrated strong performance across a range of medical image segmentation tasks. Notably, open-source models such as TotalSegmentator [2] have achieved high-quality segmentation of various anatomical structures and pathologies, setting a benchmark in the field. CNNs have also been applied successfully to liver imaging.

One study demonstrated the feasibility of automatic segmentation of liver parenchyma in multi-echo chemical shift encoded (MECSE) MRI [3]. The model showed excellent agreement with manual segmentations across both internal and external validation datasets—highlighting its robustness across scanners and vendors. However, this model was limited to whole liver segmentation and did not address focal lesion detection or segmental localization. Similarly, a study developed a deep convolutional neural network (DCNN) for automated liver segmentation for MRI [4]. The model achieved high segmentation accuracy and demonstrated robust performance. These findings underscore the model’s strong generalizability and clinical applicability for quantitative liver analysis. However, once again, the focus was on whole-liver segmentation and did not include focal lesion detection or Couinaud segment localization.

Another study proposed a deep learning approach using a deep belief network (DBN) for automatic liver segmentation in CT images, aiming to support the development of quantitative imaging biomarkers for computer-aided diagnosis [5]. While promising in terms of accuracy and computational efficiency, the approach was focused solely on liver segmentation without addressing lesion-level segmentation or anatomical localization within liver segments. Similarly, a study using Mask Region-CNN on multiphase CT data successfully detected primary hepatic malignancies, including HCC [6]. While promising, this model was limited to lesion detection without full segmentation or anatomical labeling, and it was not applied across imaging modalities.

Other work has attempted to automate LI-RADS classification by using a U-Net-based deep convolutional neural network (DCNN) to segment both the liver and HCC lesions in multiphasic MRI [7]. This study showed good performance; however, its scope remained limited to a single modality, lacked Couinaud segment localization, and was tested on a relatively small single-center dataset. A more recent study evaluated the ScaleNAS architecture, trained on multi-organ lesion datasets, for its performance in detecting and segmenting liver lesions specifically in CT imaging [8]. ScaleNAS achieved a Dice score of 70.2% in a large internal test cohort and 64.2% in an external colorectal liver metastasis (CRLM) dataset. Still, the model lacked segmental localization, MRI support, and was not optimized specifically for liver-focused pathology.

Despite these promising developments, many of the current models are constrained by modality specificity, narrow clinical focus, or absence of publicly available tools. There remains a need for a robust, openly accessible solution that supports both CT and MRI imaging, enables segmentation of both individual liver segments and lesions, and includes anatomical mapping to Couinaud segments to facilitate structured, longitudinal follow-up in clinical practice.

The aim of this study is therefore twofold: first, to update and expand upon the TotalSegmentator framework by training additional deep learning models based on the nnU-Net [9] architecture that are capable of automatically detecting and segmenting liver lesions and Couinaud liver segments on both CT and MRI; and second, to illustrate the clinical utility of these models using a representative example: the assessment of hepatocellular carcinoma (HCC) following transarterial chemoembolization (TACE). By releasing our models as open-source tools, we aim to promote broader research, enable collaborative data-sharing, and facilitate multi-institutional refinement and validation.

As such, the key contributions of this work lie in several aspects that address current limitations in liver segment and lesion segmentation. Unlike many existing models that are limited to a single imaging modality, our approach to lesion detection is trained across both CT and MRI, enhancing its applicability in diverse clinical settings. We place a strong emphasis on generalizability by demonstrating robustness across varied acquisition protocols, patient populations, and evaluating our model through an external dataset. Importantly, all models, training code, and evaluation pipelines are made openly available to encourage reproducibility, community validation, and collaborative development. Finally, we qualitatively demonstrate the model’s potential clinical utility through a longitudinal case of hepatocellular carcinoma (HCC) treated with transarterial chemoembolization (TACE), illustrating how the model could support automated lesion tracking over time. Together, these contributions position our work as a step toward more accessible, generalizable, and anatomically aware liver segment imaging tools for both clinical and research applications.

## Methods and Materials

This retrospective study was approved by the Ethics Committee of Northwest and Central Switzerland (Project ID: EKNZ BASEC 2023–00446) and was structured in accordance with the CLAIM criteria [10].

### Technical Details

CT scans were acquired using Siemens, Philips, and GE Medical Systems scanners, including the models Somatom Force, Somatom Edge, Somatom Definition Flash, Somatom Definition AS +, Somatom go.top, iCT 256, and Revolution CT, employing late arterial and portal venous phase imaging protocols, with an iodine-based contrast agent administered. The key imaging parameters—kilo Voltage peak (kVp), slice thickness, and X-ray tube current—were within the recommended ranges to optimize image quality while minimizing radiation exposure. The minimum, median, and maximum values were as follows: kVp (70 kVp, 100 kVp, 512 kVp), slice thickness (0.6 mm, 1.5 mm, 3.0 mm), and X-ray tube current (0 mA, 191 mA, 2595 mA). These protocols were specifically tailored for the transarterial chemoembolization (TACE) subset to optimize contrast enhancement and improve lesion visualization.

MRI scans were acquired using a variety of Siemens models that differed in magnetic field strength and imaging capabilities, contributing to notable variability in image quality, mirroring the real-world application of MRI. Contrast agents—including Primovist, Dotarem, and Gadovist—were administered at variable dosages, introducing further heterogeneity in tissue enhancement and lesion visibility. The primary imaging sequence, T1-weighted volumetric interpolated breath-hold examination (T1 VIBE), was used in combination with the Dixon method to improve fat suppression and signal uniformity; however, its implementation varied depending on scanner configuration and clinical considerations. Key imaging parameters—echo time, repetition time, and slice thickness—varied widely across scans, with some exhibiting extreme outliers. The minimum, median and maximum values were as follows: echo time (1.23 ms, 2.39 ms, 90 ms), repetition time (3.95 ms, 6.64 ms, 1100 ms), and slice thickness (1.5 mm, 3 mm, 6 mm), respectively. Gradient Recalled Echo (GRE) was the predominant scanning sequence, though additional protocols were selectively applied based on clinical needs. To mitigate these effects of variability of image quality, we deliberately curated an exceptionally heterogenous dataset.

#### Overview

We trained two sets of nnU-Net models: (1) “anatomical models”, which segment the whole liver on the level of the individual Couinaud segments; and (2) “lesion models”, which detect and segment any type of liver lesion.

### Data Acquisition

We performed a retrospective, diagnostic imaging study. Relevant cases were identified using structured query language (SQL) based on radiology reports and procedural codes. We then retrieved the corresponding CT and MRI images, which were loaded onto the open-source web-based platform NORA [11] for further annotation and processing. Unless otherwise stated, poor image quality, missing sequences, or incomplete data served as exclusion criteria across all cohorts.

### Liver Anatomy Segmentation Cohorts

**Fig. 1:**
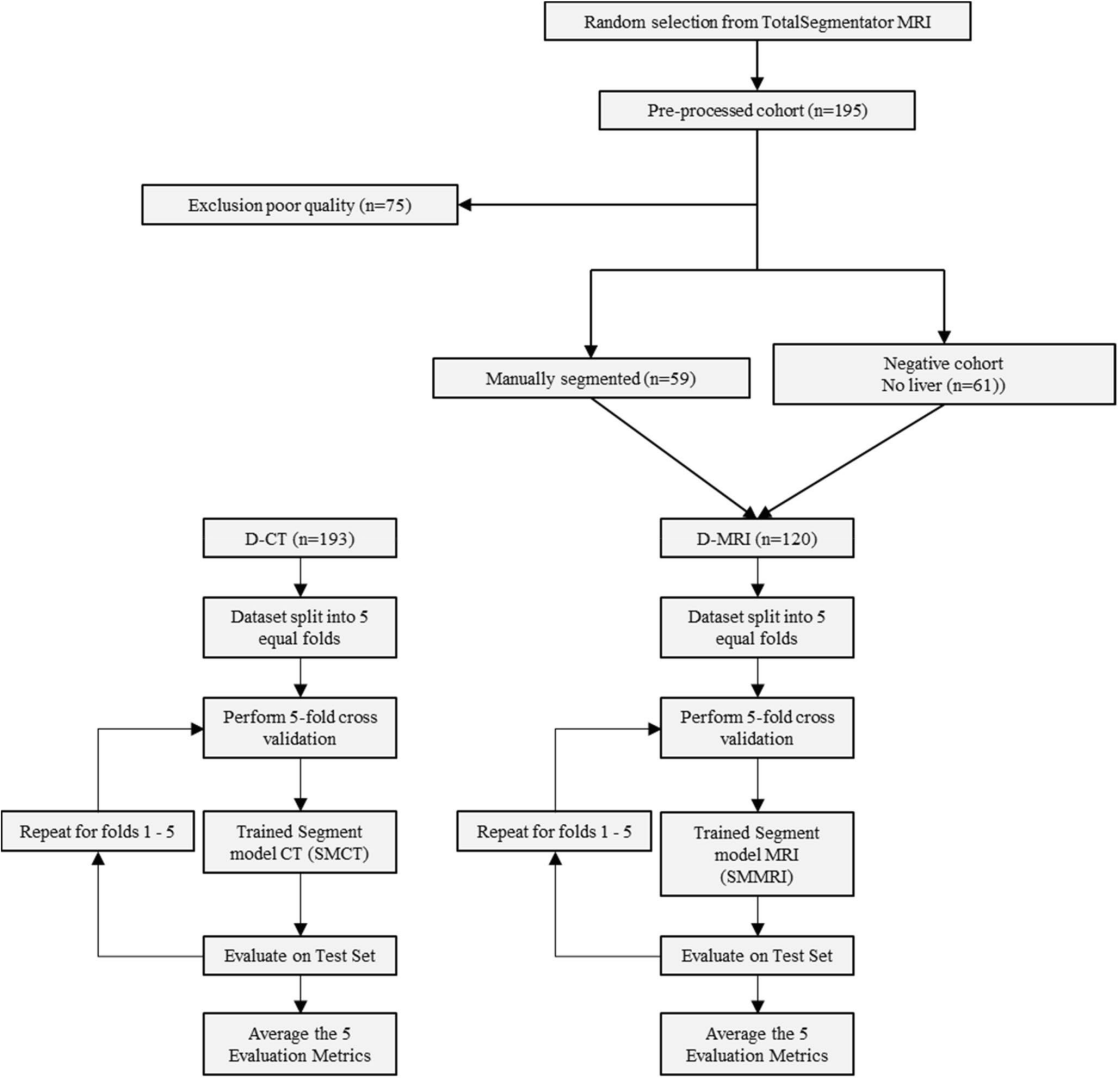
Flow chart outlining data selection and subsequent progression for liver segments

We assembled two distinct datasets for the identification and anatomical segmentation of the liver segments. The first model (SMMRI) was trained on a dataset of MRIs only, labelled “D-MRI”, derived from a subset of data employed in the TotalSegmentator MRI Project [2], where “abdomen” and “liver” served as inclusion criteria. This included 120 MRI examinations, and 59 of these, recorded between 2014 and 2022, were manually segmented by a junior physician (reader 1) under the supervision of a radiologist with 3 years of experience (reader 2). The remaining 61 MRI scans, which did not include a liver in the image, were included as a negative cohort. The second was based on a publicly available set of 193 CT scans with public annotations of the liver segments and labelled “D-CT” (CT only) [12], for training a dedicated Segment Model on CT (SMCT). Following model training, both the MRI-only model from D-MRI and the CT-only model from D-CT were tested on 120 MRIs and 193 CTs, respectively (Fig. 1).

### Lesion Detection Cohort

**Fig. 2:**
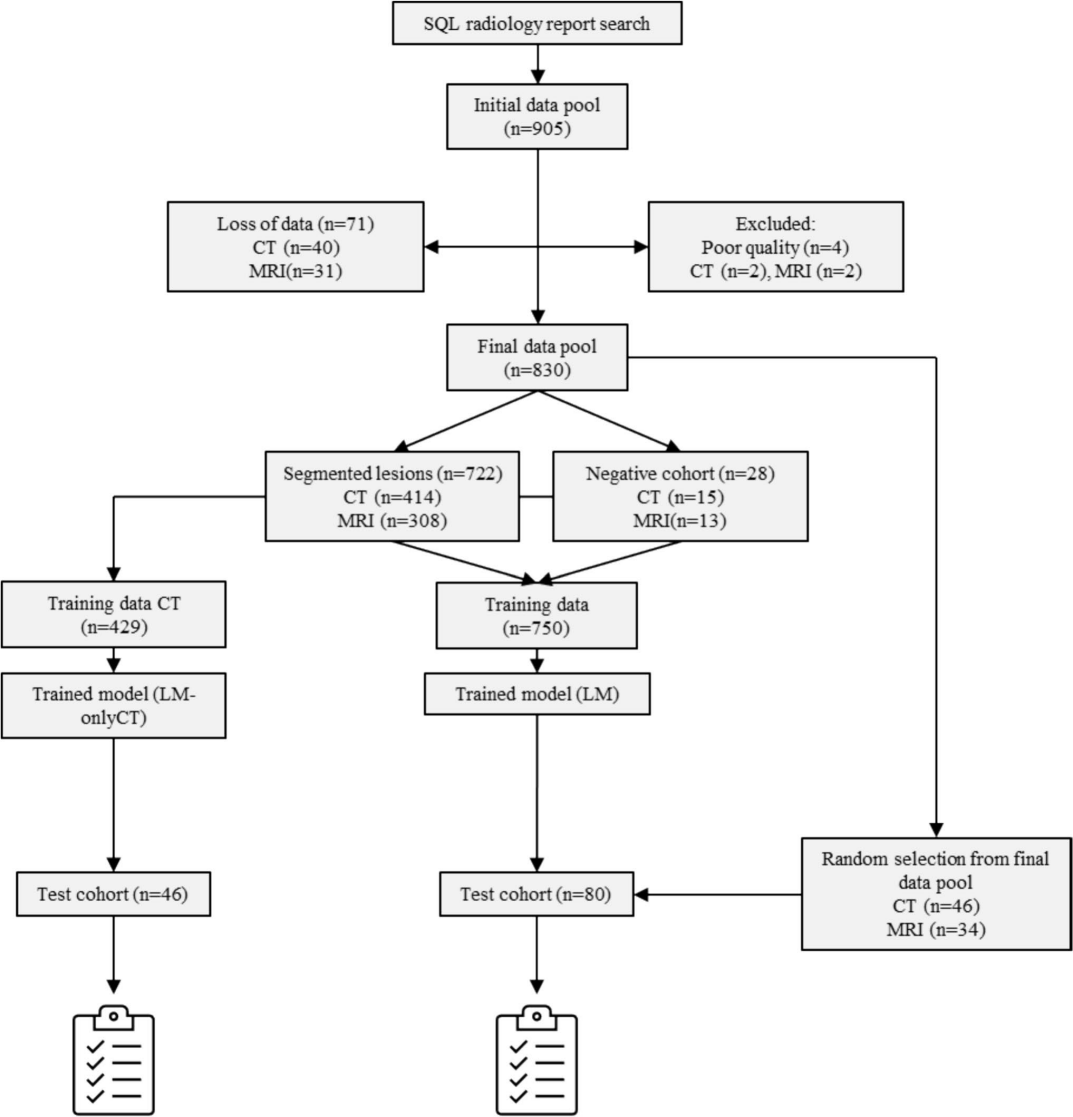
Flow chart outlining data selection and subsequent progression for liver lesions

Initially, all radiology reports corresponding to CT or MRI examinations that contained the keyword “liver” were extracted from the clinical data warehouse (cDWH). The retrieved report texts were then truncated to ± 200 characters around the keyword “liver” to focus on the relevant liver-finding sections. Prior to analysis, all text segments were de-identified to remove any potentially sensitive information of Protected Health Information (PHI). The structured reports did not contain direct identifiers, and only the liver-findings segments were retained. The truncated and anonymized text segments were subsequently screened using a large language model (GPT-4o, OpenAI, San Francisco, CA, USA) [13] to identify any mention of liver lesions including hepatocellular carcinoma (HCC), cholangiocarcinoma (CCC), abscess, cyst, haemangioma, metastasis/metastases, adenoma, focal nodular hyperplasia (FNH), cystadenoma, angiosarcoma, fibrolamellar carcinoma, hamartoma, Meyenburg complex, regenerative nodule, angiomyolipoma, and echinococcosis. This resulted in an initial cohort of 905 image studies, of which 71 were lost due to unavailable data and four studies (two CTs, two MRIs) were excluded due to poor image quality, reducing the study size to 830. We identified 750 imaging studies (429 CTs, 321 MRIs) for lesion detection training. Of these, 722 (414 CTs, 308 MRIs) were manually segmented by reader 1. An additional 28 images without confirmed lesions were included as a negative cohort to refine detection thresholds. This dataset was labelled “D-lesions”. The remaining 80 image studies (46 CTs and 34 MRIs), containing a total of 153 and 118 lesions respectively, were retained for Lesion Model (LM) testing (Fig. 2).

### TACE Cohort

Analogous to the lesion detection process, all radiology reports of CT examinations containing the keyword “TACE” were extracted from the cDWH. Swiss Classification of Surgical Procedures (CHOP) codes were extracted from the data warehouse to determine the exact date of each TACE procedure. Pre- and post-TACE CT examinations were then manually identified by matching each CT report date against the known TACE procedure date, using a comprehensive list of all CT examinations with reports. Only CT examinations were considered for the “D-TC” dataset. Inclusion required that each study fall within 6 months prior to TACE and up to 12 months afterward, allowing pre- and post-procedural comparisons. When late-arterial phase imaging [14] was unavailable, portal-venous phase scans were substituted. For patients undergoing multiple TACE procedures, post-procedural scans of one TACE could serve as the baseline (pre-TACE) for a re-TACE if no new scan was obtained. An initial 232 CT studies were retrieved; after removing incomplete or irrelevant studies, 172 were deemed valid. These final scans were used to evaluate the Lesion Model’s performance in quantifying attenuation and volume changes in HCC lesions post-TACE.

#### Segmentation

**Fig. 3:**
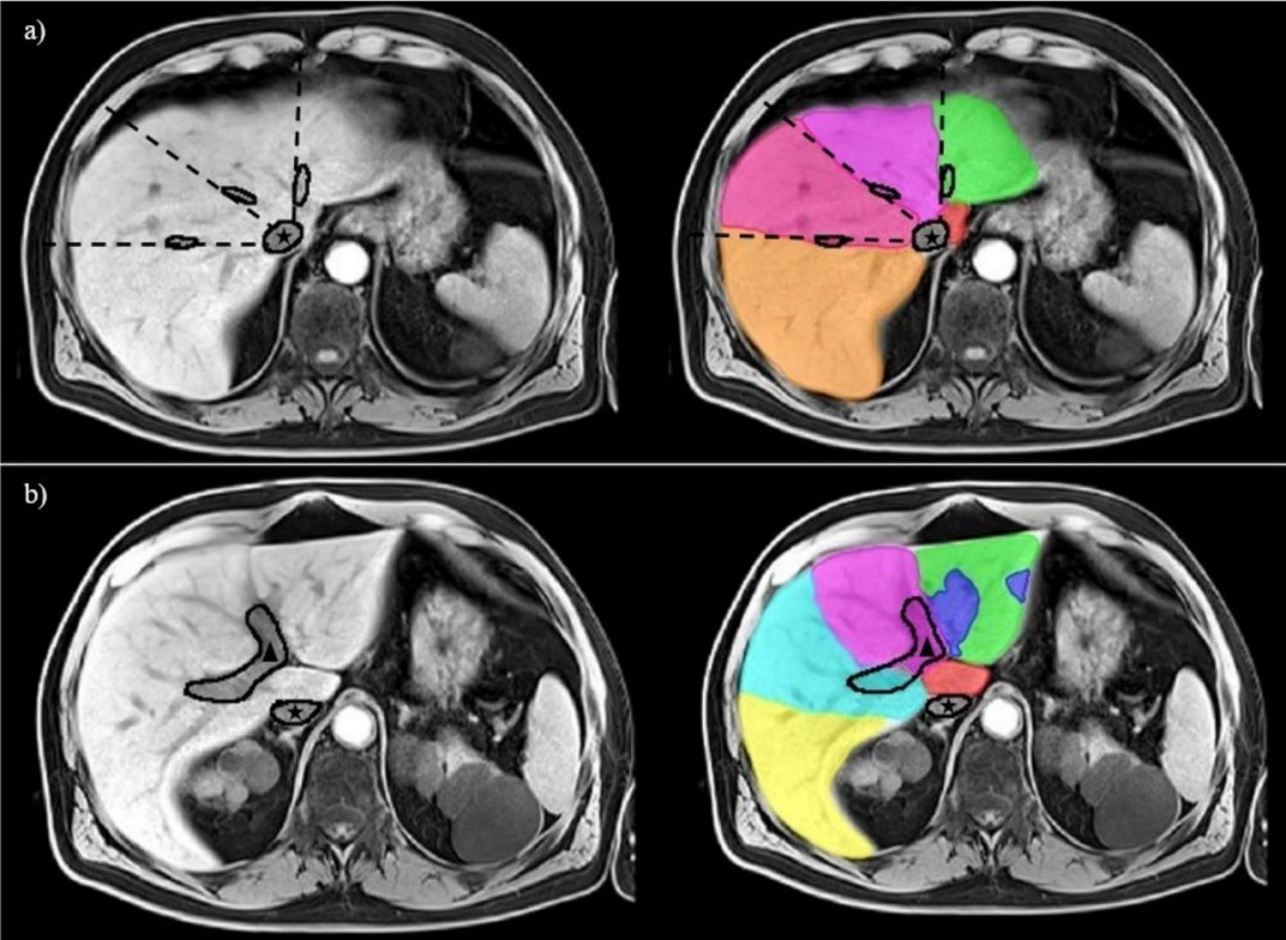
MRI cross-section of the liver at different slice positions: Star indicating the IVC, triangle indicating the bifurcation of the portal vein, crosscut of dotted line through the hepatic veins (plain encircled) to the IVC, delineating the liver segments. Clockwise in **a** S 7,8,4,2,1—orange, magenta, pink, green, red, and **b** S 6,5,4,3,2,1—yellow, cyan, pink, blue, green, red

Reader 1 performed voxel-wise liver segment segmentation based on Couinaud’s classification [15] while being supervised by reader 2. The hepatic veins serve as vertical dividers of the liver into segments (S) 7,8,4,2 from the superior clockwise, running through the inferior vena cava (IVC), with the portal vein (PV) acting as the reference point for the horizontal division of the liver and signalling the turnover to S 6,5,4,3. Segment 1 lies anteromedial to the IVC. Figure 3 illustrates the anatomical structures used as reference points for the segmentation process. The reader 2 supervision approach was used to generate the reference standard for liver segmentation.

Lesion segmentation employed the same general approach. Reader 1 manually segmented 722 imaging studies, excluding four poor-quality examinations after additional review. The manual segmentation was performed on a voxel-wise basis, delineating lesion boundaries slice-by-slice in three dimensions to generate volumetric annotations. Reader 2 provided supervision to Reader 1, resulting in the correction of 12 CTs and 19 MRIs and exclusion of cases with low image quality or with complex postoperative anatomy (*n* = 4). This process was conducted using the NORA [11] medical imaging platform, a web-based framework designed for visualizing, organizing, processing, and sharing medical imaging data. These manually delineated annotations served as the ground truth reference standard for evaluating subsequent automated segmentation performance, following a methodology similar to that described in previous work [16], where manual segmentation provides reliable reference data for validating automated methods.

### Model Training and Evaluation

**Fig. 4:**
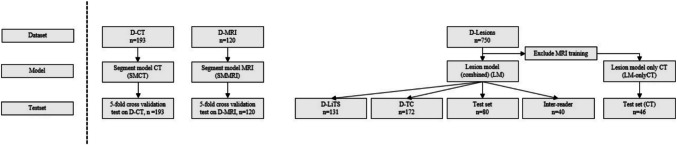
Flowchart depiction of datasets allocated to model and test sets

We used the nnU-Net framework [9] to train four separate models. No additional preprocessing was performed, as nnU-Net includes a self-configuring pipeline that automatically handles key preprocessing steps such as resampling, normalization, and cropping. This built-in functionality ensures standardized and consistent input preparation across imaging modalities and datasets, promoting model generalizability. The framework’s design underscores the importance of preprocessing in medical image segmentation and offers a user-friendly approach that can yield significant performance benefits—an observation also emphasized by Ansary et al. [17] in their work on the impact of data preprocessing on model performance. The Segment Model MRI (SMMRI) was trained on the dataset D-MRI, while the Segment Model CT (SMCT) was trained on D-CT. The segment models were tested on the datasets using fivefold cross validation. The Lesion Model (LM) was trained and tested on the dataset D-Lesions, using an iterative optimization process, as described in [2]. To evaluate the impact of MRI data, a second model restricted to CT images alone (LM-onlyCT) was also trained using D-Lesions excluding MRI data. Following these training steps, the LM was applied to D-TC as a qualitative example to illustrate the model’s ability to detect and segment HCC lesions across pre- and post-TACE timepoints (Fig. 4).

Lesion detection required at least 20% overlap in volume between the model’s prediction and the reference segmentation, and an absolute lesion volume of at least 0.06 ml. Volume differences indicate the degree of deviation between the ground truth and the predicted segmentation. A positive volume difference means the segmented volume in the ground truth is greater than the prediction, whereas a negative difference indicates the predicted volume exceeds the ground truth. A threshold of 15 lesions per scan was set to avoid disproportionate metrics in patients with disseminated disease; if more than 15 lesions were found, detection was recorded and classified as 1 (i.e., fully detected) in the sensitivity metric. To assess inter-rater agreement, 40 cases (20 CTs, 20 MRIs) were randomly chosen from the training data. Both readers segmented these studies independently and were blinded from the other reader’s segmentation, enabling a comparison of each reader’s performance against the other as well as against the model. Finally, to assess the generalizability of our model, we evaluated its performance on the publicly available Liver Tumor Segmentation (LiTS) dataset [18]. This dataset consists of contrast-enhanced abdominal CT scans with expert-annotated liver lesions, providing an external benchmark for lesion detection and segmentation. We applied our trained model directly to the LiTS test data without additional fine-tuning and computed standard detection and segmentation metrics to compare performance.

### Statistical Analysis

Primary performance metrics included lesion wise sensitivity, false positives per case (FP/c), Cohen’s kappa measurement for detection, and the Dice coefficient for segmentation overlap. Additional metrics, such as the positive predictive value (PPV), the F1 score, precision, and volume change supplemented detection and segmentation evaluations. Model comparison was conducted by evaluating for significant differences and *p*-values. To evaluate treatment response in the TACE dataset (TC), both Hounsfield Unit (HU) attenuation changes and volumetric differences were calculated by comparing pre- and post-procedure contrast-enhanced CT scans. Tumor regions were segmented at both time points, and median HU values were extracted from the segmented tumor volumes. Attenuation change was defined as the difference between post- and pre-TACE median HU values. Tumor volume was computed by multiplying the number of voxels within each segmented lesion by the voxel volume. For each case, percentage changes in both HU and volume were calculated relative to the pre-TACE baseline. To summarize these results across the cohort, we report the median and interquartile range (IQR) of the HU and volume changes. False positives per case (FP/c) were included as a metric to support the model’s utility as a tool for disease screening. Ninety-five percent confidence intervals were calculated using a non-parametric bootstrapping approach with 1000 iterations. Normality was tested using the Shapiro–Wilk method; comparisons employed the two-sided Wilcoxon signed-rank test, with *p* < 0.05 defining statistical significance.

## Results

### Performance of Anatomical Liver Segment Segmentation

**Fig. 5:**
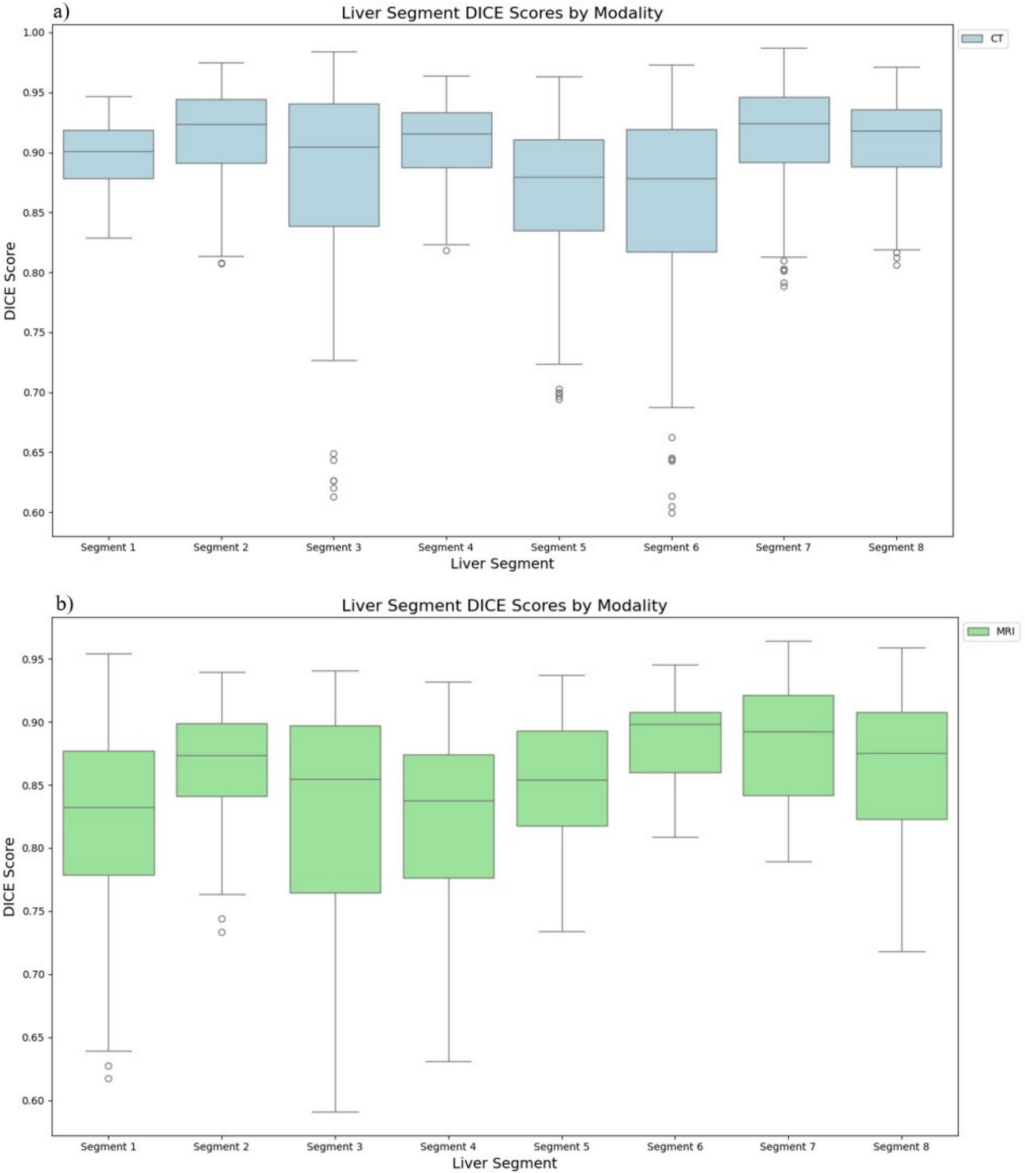
Boxplots illustrating the performance of liver segment segmentation across different liver segments, with the Dice similarity coefficient (Dice score) on the y-axis and the liver segments on the x-axis. **a** Segmentation results for the CT modality. The distribution of Dice scores across all liver segments demonstrates generally high performance, with a mean Dice score of 0.897 (95% CI: 0.894–0.900). The boxplots highlight both the central tendency and the variability in performance across segments, with relatively small interquartile ranges indicating consistent segmentation quality. **b** Segmentation results for the MRI modality. Compared with CT, the Dice scores are lower overall, with a mean Dice score of 0.847 (95% CI: 0.839–0.855). The boxplots show more pronounced variability across different segments, reflecting greater challenges in MRI-based segmentation. Together, the two panels emphasize that while segmentation is robust in both modalities, CT achieves more consistent and higher Dice similarity across liver segments than MRI

**Fig. 6:**
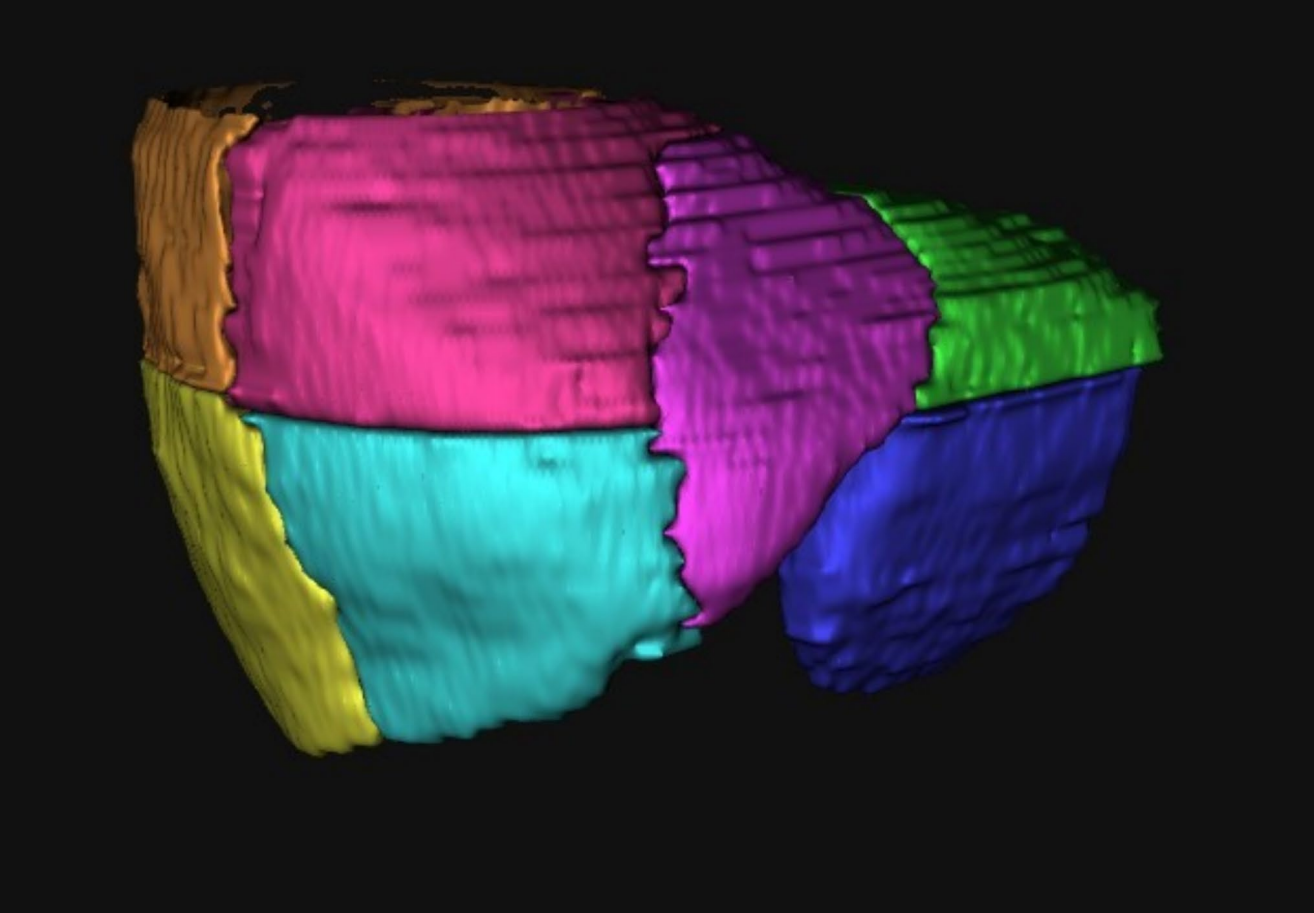
Example of a 3D model of the liver illustrating automated liver segmentations. The individual Couinaud segments are color-coded for visual distinction and are presented in clockwise order: segment 2 (green), segment 3 (blue), segment 4 (pink), segment 5 (cyan), segment 6 (yellow), segment 7 (orange), and segment 8 (magenta). Segment 1 is not depicted, as it is located posteriorly and not visible in the planar orientation of this rendering. This figure provides a qualitative example of the segmentation model output and highlights the ability of the method to delineate liver anatomy into distinct functional segments

The SMCT achieved a mean Dice similarity coefficient of 0.897 (95% CI: 0.894–0.900) across 193 CT examinations, while the SMMRI achieved a mean Dice score of 0.847 (95% CI: 0.839–0.855) across 120 MRI examinations. These values represent the mean segmentation performance across individual liver segments. Figure 5 illustrates the box-plot visualization of these results for each modality. Figure 6 shows an example of a wholly automatically segmented liver delineated according to its anatomical segments.

### Performance of Liver Lesion Detection

**Fig. 7:**
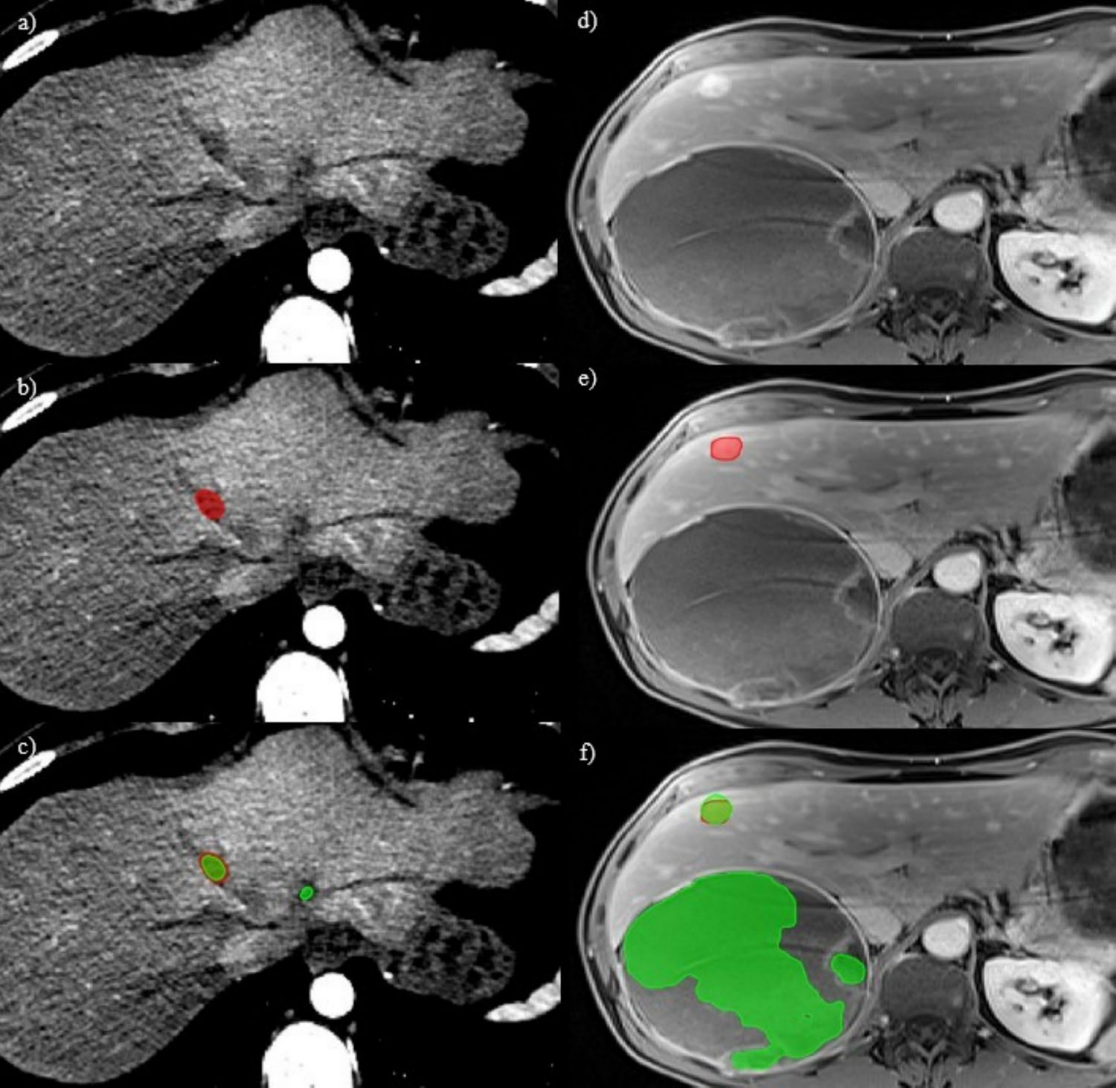
Cross-sectional examples in CT and MRI demonstrating both correct and incorrect model segmentation outcomes. **a**–**c** CT modality: **a** Original CT slice; **b** ground-truth annotation of a hemangioma in liver segment IV (red); and **c** model segmentation overlay, which correctly identifies the hemangioma but also produces a false positive by misclassifying a blood vessel as a lesion (green). **d**–**f** MRI modality: **d** Original MRI slice; **e** ground-truth annotation of a hemangioma in liver segment V (red); and **f** model segmentation overlay, which again captures the true lesion but incorrectly identifies a kidney tumor as a false positive (green). These examples demonstrate both the strength of the model in detecting true hepatic lesions across modalities and the challenge of avoiding false positives, particularly when nearby vessels or extrahepatic structures mimic lesion characteristics

The LM evaluated on CT + MRI achieved a sensitivity of 70.1% (95% CI: [0.625,0.775]), a FP/c of 0.738 (95% CI: [0.388,1.100]), and a Dice score of 0.522 (95% CI: [0.431,0.611]). The LM achieved a sensitivity of 75.8 (95% CI: 66.7–83.7), a FP/c of 0.522 (95% CI: 0.196–1.043), and a Dice similarity coefficient of 0.658 (95% CI: 0.568–0.761) on CT. On MRI, it achieved a sensitivity of 62.7 (95% CI: 47.2–73.4), FP/c of 1.029 (95% CI: 0.441–1.706), and a Dice coefficient of 0.337 (95% CI: 0.211–0.464). The positive predictive values (PPV) of the LM on CT and MRI were 82.9% and 67.9%, respectively. The LM-only CT demonstrated a sensitivity of 72.6 (95% CI: 62.8–81.6), an FP/c of 0.500 (95% CI: 0.261–0.804), and a Dice coefficient of 0.632 (95% CI: 0.533–0.739). In a comparison of the model trained on both MR and CT data with the model trained solely on CT data, no statistically significant difference was observed (*p* = 0.05). Nevertheless, we chose the MR + CT model for further use due to its higher sensitivity and the benefit of being trained on more diverse imaging data, which we anticipate will improve its generalizability. On the LiTS test set (131 lesions), our model achieved a sensitivity of 65.7% (95% CI: 55.5%–74.1%), a FP/c of 0.73 (95% CI: 0.50–0.95). For segmentation quality, the model achieved a Dice score of 0.59 (95% CI: 0.54–0.65). The PPV was 80.17%. A comparison of the CT model subsets is detailed in Table 1. Figure 7 shows an example of the automatic segmentation of liver lesions in CT and MRI, including an example of false positive segmentation.

**Table 1:** Comparison of the subset models on CT with the primary evaluation metrics used and comparative metrics

	CT (LM)	CT (LM-only CT)
Sensitivity	75.8 (95% CI: [66.7, 83.7])%	72.6 (95% CI: [62.8,81.6])%
Dice Score	0.658 (95% CI: [0.568, 0.761])	0.632 (95% CI: [0.533, 0.739])
FP/c	0.522 (95% CI: [0.196, 1.043])	0.500 (95% CI: [0.261, 0.804])
Volume difference	−1.519 (95% CI: [−16.254, 11.907]) ml	−2.223 (95% CI: [−16.405, 9.838]) ml
*p*-value	0.05	
Difference	Non-significant difference

### Inter-reader Performance vs. Model

In a subset of 40 studies, the inter-reader performance yielded a sensitivity of 64.7 (95% CI: 0.525–0.760), an FP/c of 0.775 (95% CI: 0.300–1.375), and a Dice score of 0.464 (95% CI: 0.340–0.582) for reader 1 vs. reader 2. The performance of reader 1 vs. LM yielded a sensitivity of 53.2 (95% CI: [0.431,0.654]), an FP/c of 0.825 (95% CI: [0.325,1.450]), and a Dice score of 0.432 (95% CI: [0.314,0.546]). The calculated Cohen’s kappa coefficient to assess inter-rater agreement was 0.244**.** The volume difference of 3.864 ml (reader 1 vs. LM) and –0.108 ml (reader 1 vs. reader 2) and indicate that reader 2 segmented slightly more of the lesions than reader 1 whereas the Model segmented less than reader 1; however, these differences are not significant, as they fall within the confidence interval (Table 2).

**Table 2:** Summary of key metrics (sensitivity, FP/c, and DICE score) across various evaluation types. It shows the model’s (LM) performance compared to different readers

	reader 1 vs. LM	reader 1 vs. reader 2
Sensitivity	53.2 (95% CI: [43.1, 65.4]) %	64.7 (95% CI: [52.5, 76.0]) %
DICE-Score	0.432 (95% CI: [0.314, 0.546])	0.464 (95% CI: [0.340, 0.582])
FP/c	0.825 (95% CI: [0.325, 1.450])	0.775 (95% CI: [0.300, 1.375])
Volume difference	3.864 (95% CI: [− 3.687, 14.971]) ml	− 0.108 (95% CI: [− 5.004, 4.305]) ml

### Performance of TACE Lesion Segmentation

**Fig. 8:**
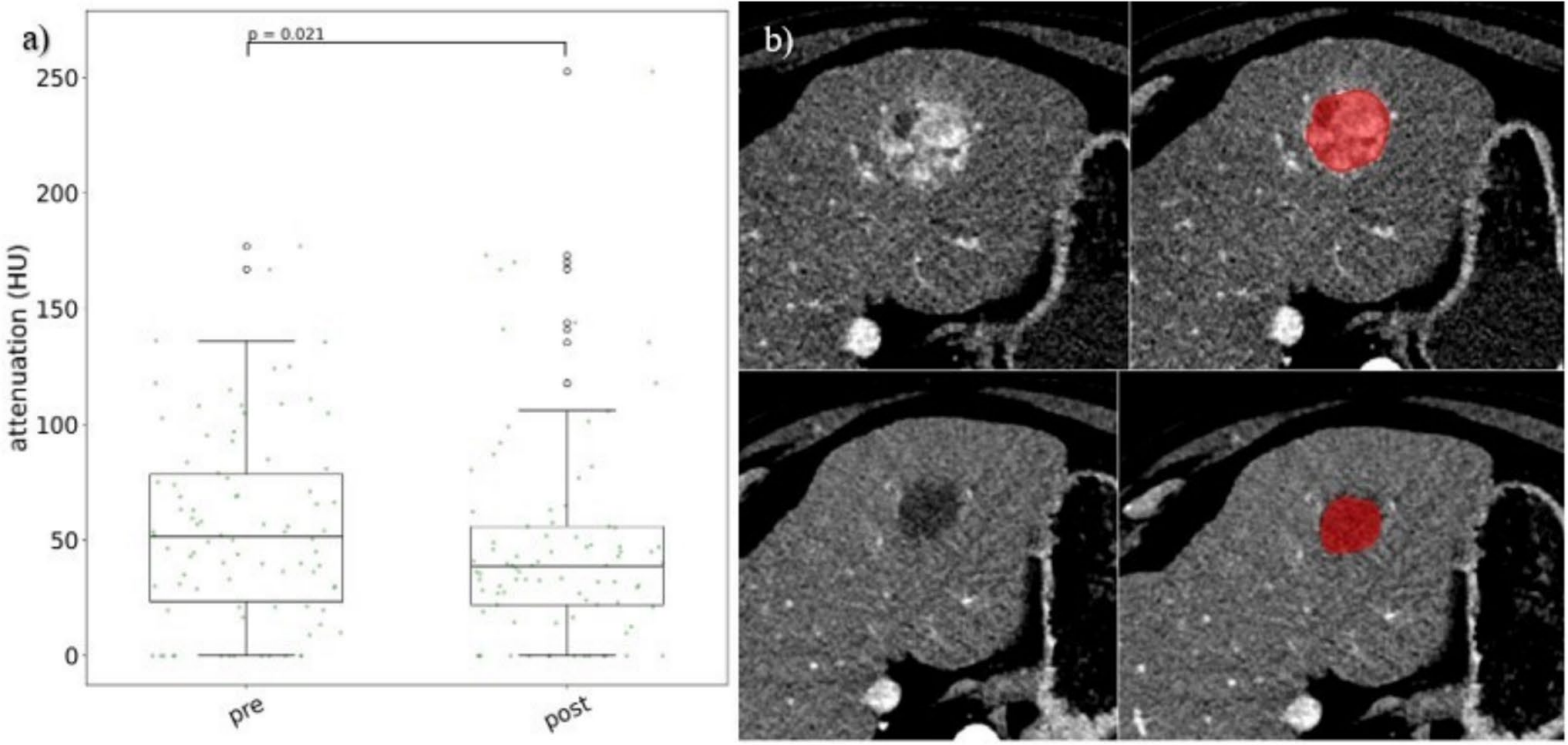
Side-by-side boxplot analysis of attenuation changes in a segmented hepatocellular carcinoma (HCC) lesion before and after a transarterial chemoembolization (TACE) procedure. **a** The median attenuation value decreased from 51.33 HU pre-TACE to 38.50 HU post-TACE, a statistically significant change (*p* = 0.021). **b** Lesion segmentation images, with the upper row showing the pre-TACE scan and the lower row showing the post-TACE scan, illustrating the segmented tumor region at both timepoints. Together, these panels demonstrate how automated segmentation can be used not only for accurate lesion localization but also for quantitative radiological assessment, enabling objective evaluation of treatment response following TACE

The LM showed a statistically significant decrease in HCC attenuation (*p* = 0.021); the median attenuation value was 51.33 HU (IQR of 23.38–78.50 HU) pre-TACE and 38.50 (IQR of 22.00–55.88 HU) post-TACE. The volume recorded a non-significant increase in volume (*p* = 0.32) following TACE. The median volume was 2.41 ml (IQR of 0.247–9.23 ml) pre-TACE and 4.07 ml (IQR of 0.658–11.13 ml) post-TACE. The reduction in HCC attenuation is visualized in the boxplot below (Fig. 8a), including an example of a HCC pre- and post-TACE with and without model (LM) annotation (Fig. 8b).

## Discussion

In this study, we developed and validated deep-learning models based on the nnU-Net framework to detect and segment both liver anatomy (Couinaud segments) and a wide variety of liver lesions on a heterogeneous dataset of both CT and MRI. Our results demonstrated robust and reliable segmentation performance across individual liver segments (mean Dice ~ 0.897 for CT and ~ 0.847 for MRI) and encouraging accuracy in identifying diverse liver lesions (CT sensitivity 75.8%, Dice 0.658; MRI sensitivity 62.7%, Dice 0.337). These findings illustrate the potential for these models to aid the radiologist with tedious tasks such as lesion volume analysis and attenuation.

This study demonstrated the clinical feasibility of employing these models for the automated detection, segmentation, and volumetric assessment of liver lesions in patients with hepatocellular carcinoma, both prior to and following TACE treatment, focusing on changes in attenuation and lesion volume. This served as a robust case study for a broader range of possible automated analyses, spanning tumor volumetry, disease burden, or liver remnant volume in both benign and malignant lesions. These models therefore have the capacity to streamline imaging workflows by providing rapid, reproducible volumetric assessments and assisting radiologists with these tedious tasks. These capabilities can become especially relevant for follow-up protocols and for future projects exploring additional response criteria, such as RECIST [19] or emerging quantitative biomarkers. Moreover, these quantitative measures—especially volumetry and attenuation—could integrate with established guidelines like the Liver Imaging Reporting and Data System (LI-RADS) [20] and modified RECIST (mRECIST) [19], both of which leverage lesion features to characterize and monitor HCC and malignancies, respectively. Automated output might improve the consistency of parameters required for such frameworks, thereby strengthening clinical decision-making and enabling more nuanced comparisons across time and institutions.

Our model achieved Dice similarity coefficients of 0.897 for CT and 0.847 for MRI liver segmentation, demonstrating strong and consistent performance across modalities. Peijun Hu’s [21] method, which focused exclusively on CT liver segmentation using the SLIVER07 dataset, reported a high mean Dice score of 0.9725, reflecting excellent accuracy likely supported by their combined 3D CNN and surface optimization approach for whole liver segmentation. For a more precise comparison, Yinli Tian et al. [22] developed a fully automated method for Couinaud liver segment segmentation on CT images using a deep neural network with spatial adaptation. Their approach focuses on detailed anatomical subdivision of the liver into functional segments. Similarly, our CT model performs individual liver segment segmentation, achieving a Dice similarity coefficient of 0.897 compared to 0.882 reported by Yinli Tian et al. This demonstrates strong and comparable performance in segment-level delineation. While Tian et al.’s method leverages spatial adaptation mechanisms to enhance segmentation accuracy, our model achieves robust results relying solely on the nnU-Net automated framework.

In contrast, Xinjun Han’s method [23], developed for MRI liver segmentation, compared to the 3D-CNN model described by Han et al., which achieved a Dice score of 0.920 for liver segment segmentation on portal venous phase MRI, our nnU-Net–based approach attained a DSC of 0.847, indicating robust performance in the inherently more challenging MRI domain. Unlike the 3D-CNN, which was trained and evaluated exclusively on a single, relatively homogeneous imaging phase, our model was trained on a broader set of MRI data without prior selection of acquisition phase. This introduced greater variability in image contrast and anatomy, making the segmentation task more challenging but enhancing the model’s applicability to diverse real-world clinical settings.

Thus, while our CT segmentation accuracy surpasses that of Yinli, and our MRI results are reasonably close to Xinjun Han’s MRI-based approach, our model shows effective generalization across both imaging modalities. This highlights its versatility and practical utility for liver segmentation in diverse clinical imaging settings. Together, these findings support the feasibility of accurate automated liver segment segmentation, with potential for further improvements by integrating advanced spatial modeling techniques.

Meanwhile, lesion-detection approaches have reported higher sensitivity when addressing a single lesion subtype, a narrower imaging scope such as specific MRI protocols, defined cohorts (e.g., patients with colorectal cancer liver metastases), or subdividing into size categories, which may limit generalizability.

In the study by Vorontsov et al. [24], a fully convolutional network (FCN) was evaluated for automated detection and segmentation of colorectal liver metastases (CLMs) on contrast-enhanced CT. The FCN demonstrated detection sensitivities of 10%, 71%, and 85%, and positive predictive values (PPVs) of 25%, 83%, and 94% for lesions < 10 mm, 10–20 mm, and > 20 mm, respectively. In contrast, our nnU-Net CT model achieved an overall sensitivity of 75.8% and PPV of 82.9%, which indicates comparable performance to the FCN for mid-sized lesions, somewhat lower PPV for large lesions, but—crucially—far superior sensitivity and PPV when small lesions are considered. Since small lesions (< 10 mm) are notoriously difficult to detect, our model’s strong overall metrics reflect a more balanced and robust detection capability across lesion sizes, suggesting improved clinical utility in identifying smaller metastases without compromising precision.

For MRI, Dai et al. [25] produced sensitivity of 88.3% for combined AI and radiologist versus radiologist alone performance. However, their study applied much narrower lesion inclusion criteria, in contrast to our much more inclusive and heterogenous dataset. Our more general-purpose design deliberately embraced heterogeneous datasets (often postoperative) with minimal exclusion criteria, allowing our models to address a multitude of radiological scenarios at the cost of some performance trade-offs. These differences must be acknowledged whenever comparing performance across studies with varying methodologies and training sets.

Finally, evaluating our model on the publicly available LiTS dataset [18], we achieved a sensitivity of 65.7% (95% CI: 55.5%–74.1%) and a Dice coefficient of 0.593 (95% CI: 0.543–0.651) over 131 lesions. These results indicate that our model detects approximately two-thirds of the lesions with a reasonable degree of overlap between predicted and ground truth segmentations. Compared to published LiTS challenge results, where top-performing methods report Dice scores typically ranging from 0.67 to 0.74 and lesion-wise sensitivity between 46 and 63%, our model’s sensitivity is competitive and slightly higher than many earlier approaches, although the Dice score is somewhat lower than the highest-performing methods. This suggests that while our model effectively identifies lesions, there remains room for improvement in accurately delineating lesion boundaries. Overall, these findings demonstrate that our model generalizes well to external data and performs comparably to established approaches in lesion detection and segmentation. In comparison to other approaches evaluated on the LiTS dataset, our nnU-Net-based pipeline represents a more streamlined and general-purpose solution, yet it performs below more specialized, multi-step methods.

For example, the method proposed by José Denes Lima Araújo et al. [26] combines RetinaNet for initial lesion detection with U-Net for refined segmentation, achieving a Dice coefficient of 0.83 and a sensitivity of 83.9%. In contrast, our model yielded a Dice coefficient of 0.593 and a sensitivity of 65.7%. Their pipeline reflects a more evolved, tailored process that explicitly separates detection and segmentation tasks—likely contributing to improved lesion localization and accuracy. While our approach benefits from automation and simplicity without dataset-specific tuning, these results highlight the trade-off between generalizability and task-specific optimization. Future work may benefit from incorporating dedicated detection components or multi-stage architectures to bridge this performance gap. As a final illustrative example of the model’s potential clinical applicability beyond static image analysis, we examined its performance in a longitudinal setting involving treatment response. While the primary validation of our model is based on both internal and external datasets, we include a qualitative example from a cohort who underwent transarterial chemoembolization (TACE) to illustrate the potential of our model to support automated analysis in longitudinal clinical workflows. As shown in Fig. 8, the model is able to segment the lesion both pre- and post-TACE, highlighting measurable changes in lesion volume and attenuation. We emphasize that this example is not used as part of the quantitative evaluation, and no claims of agreement with human assessments are made here. Instead, it serves to demonstrate how the model’s outputs could, in future work, support follow-up assessments or treatment monitoring. A more rigorous evaluation of the model’s ability to accurately capture lesion changes in alignment with expert assessments is needed to further establish its utility in treatment monitoring scenarios.

Regarding our work: the first task of segmenting the liver according to the Couinaud segments was undertaken to enhance the TotalSegmentator project by adding detailed anatomical segmentation capabilities. This work served as a foundational step and a springboard into the second task of lesion detection and segmentation, enabling a more comprehensive liver analysis framework. The decision to develop separate models for Couinaud liver segmentation on CT and MRI, but a joint model for lesion segmentation, was based on observed performance differences. Liver segmentation models trained individually on CT and MRI achieved satisfactory and literature-comparable results, indicating that modality-specific models were sufficient. In contrast, lesion segmentation was performed using a single joint model trained on both CT and MRI data, reflecting our philosophy of developing generalized and easy-to-use tools that maximize performance while maintaining simplicity and broad applicability. Thus, the joint model was adopted for lesions to align with this approach, while separate models were used for the more consistent liver segmentation task. Although a total multimodal fusion approach combining features from both CT and MRI may offer potential benefits by leveraging complementary information, this was not pursued in the current study due to practical considerations and the satisfactory performance of the chosen modeling strategies. Exploring such fusion techniques represents a promising direction for future work.

Although our findings demonstrate that the nnU-Net–based approach can approximate the performance of a radiologist for CT lesion detection, there remain important caveats. Lesions with highly variable appearances, suboptimal image quality or contrast phases—particularly in MRI, due to differences in field strength, imaging parameters, contrast agents, and their dosages—as well as operator-dependent segmentation, continue to present significant challenges. This difficulty is reflected in our Cohen’s kappa analysis of inter-rater agreement between two readers, which yielded a kappa of 0.244, indicating fair agreement. This underscores the inherent ambiguity and subjectivity in lesion identification, suggesting that variability between human readers should be considered when comparing automated methods to manual annotations. Given this, the automated approach’s performance relative to human readers is encouraging.

A particular limitation in medical image segmentation of Couinaud liver segments stems from the liver’s anatomical complexity and the natural variability in size, shape, and frequency among its segments. Smaller regions, such as segments I and IVa, are often underrepresented compared to larger, more prominent segments like V or VIII. This results in a pronounced class imbalance that can bias deep learning models toward the majority regions, ultimately reducing segmentation accuracy for the less frequent or smaller segments. The nnU-Net framework offers a general-purpose solution by dynamically adapting to dataset-specific characteristics through strategies such as patch-based sampling, loss function weighting (e.g., Dice or focal loss), and robust data augmentation. Moreover, using a diverse dataset encompassing variations in liver morphology, pathologies, and imaging modalities can further enhance generalization and reduce imbalance-related performance disparities. However, while nnU-Net provides a strong baseline, its automated nature may not fully resolve fine-grained segment-level imbalances in anatomically complex organs like the liver. In contrast, more tailored solutions—such as the framework described by I.A.C.M et al. [27] demonstrate how domain-specific techniques like multiscale feature aggregation and independent component analysis (ICA) can more effectively address imbalance. These methods, however, typically require increased manual input in terms of feature design and model tuning, limiting their scalability and adaptability across broader clinical segmentation tasks. While our focus in this work has been on the anatomical segmentation accuracy and clinical robustness of Couinaud liver segments using deep learning, we acknowledge that computational efficiency remains an important consideration—particularly for real-time or large-scale applications. Parallel computing approaches, such as those described in Lattice-Boltzmann-based biomedical simulation frameworks [28, 29], have demonstrated substantial acceleration in high-resolution visual processing tasks. Although such methods could potentially benefit deep learning-based segmentation pipelines, particularly in training and inference on volumetric data, integrating hardware-specific optimizations (e.g., FPGA or heterogeneous SoC architectures) would considerably broaden the technical scope of this study. We therefore consider this a promising avenue for future research, particularly in the context of deployment and real-time clinical integration.

Further, a potential limitation of this study lies in the cohort splitting strategy, which was performed at the study level rather than strictly at the patient level. This raises the possibility of data leakage, where multiple scans from the same patient may have appeared in both the training and test sets. While this can theoretically inflate performance metrics, several mitigating factors reduce its practical impact in our case. The proportion of patients with multiple scans was small relative to the overall cohort size, and the included scans often exhibited substantial variability due to treatment effects, disease progression, and natural anatomical changes over time. These factors introduce sufficient heterogeneity to reduce the likelihood that the model overfits to patient-specific features. Nonetheless, we acknowledge this as a methodological limitation and suggest that future work implement patient-level splitting and prospective validation to more rigorously assess model generalizability.

The study’s single-center design may also hinder generalizability to external populations, and direct comparisons to expert readers should be interpreted prudently.

Nonetheless, the open-source release of our models can help mitigate these factors, as broader usage and data-sharing across institutions may allow for more extensive validation, collaborative improvements, and refinements tailored to specific clinical needs. It extends and improves upon the TotalSegmentator [2] which is publicly available at the following GitHub repository [30], a model designed for automatic, sequence-independent segmentation of major anatomical structures. Our current work enhances its capabilities by incorporating updated algorithms and expanded datasets. These advancements are already partially integrated into the existing framework and will be fully incorporated in future iterations, ensuring seamless alignment with ongoing institutional initiatives.

Furthermore, it serves as a valuable starting point for further research, particularly in volumetric evaluation of the liver for preoperative surgical planning and evaluation. One such application is the automatic measurement of liver volume before and after portal vein embolisation.

By offering a flexible tool that functions despite an enormous variability of liver lesion morphology, a variety of imaging techniques and heterogeneous cases including complex postoperative cases, this study brings a publicly accessible solution for both research purposes and routine clinical practice. We believe that our contribution lies in demonstrating a robust, adaptable framework rather than focusing on a singular condition or imaging phase. As automated methods continue to evolve, they may become an invaluable adjunct in improving consistency and efficiency for liver imaging. Future investigations should develop larger multicenter cohorts, include enhancements and optimization techniques as well as deepened exploration into volumetric and functional parameters that could complement conventional size-based metrics as well as assess this tool’s ability to facilitate radiology report composition, for example through the automatic insertion of liver lesion volume in standardized mRECIST tables. These steps would further clarify the broader impact of automated segmentation tools in clinical decision-making and open new research avenues, including the integration of emerging machine learning techniques for individualized patient care.

## Conclusion

In conclusion, our approach underscores the potential of deep-learning models for cross-modality liver segment segmentation and lesion detection, while highlighting that ongoing, collaborative refinement is essential for tackling the complexity of clinical imaging data. The study’s strengths include its inclusive dataset and open-source ethos, whereas limitations revolve around single-institution sampling and inherent data heterogeneity. Nonetheless, these results mark a step toward practical, wide-ranging applications that could ultimately support radiologists in managing liver pathologies with greater efficiency and consistency.

## Data Availability

Our model is publicly available on GitHub to support further research and collaboration.
